# Kiwicha in Gastrointestinal Digestion: Structure, Bioaccessibility, Processing and Health-Relevant Functionality

**DOI:** 10.3390/foods15213826

**Published:** 2026-10-26

**Authors:** Carlos Ruiz, Gian Pierre Silvera-Otañe, Marcio Schmiele, Luz María Paucar-Menacho

**Affiliations:** 1 Programa de Doctorado en Ingeniería Agroindustrial, Mención Transformación Avanzada de Granos y Tubérculos Andinos, Universidad Nacional del Santa, Nuevo Chimbote 02712, Peru; 2025818019@uns.edu.pe (C.R.); 2025818011@uns.edu.pe (G.P.S.-O.); 2 Laboratório Integrado de Cereais e Lipídeos (LICEL), Instituto de Ciência e Tecnologia (ICT), Universidade Federal dos Vales do Jequitinhonha e Mucuri (UFVJM), Diamantina 39100-000, Brazil; marcio.sc@ict.ufvjm.edu.br; 3 Departamento Académico de Agroindustria y Agronomía, Facultad de Ingeniería, Universidad Nacional del Santa, Nuevo Chimbote 02712, Peru

**Keywords:** *Amaranthus caudatus*, bioactive compounds, food matrix, food processing, protein hydrolysis, starch digestibility

## Abstract

Kiwicha (*Amaranthus caudatus* L.) is an Andean pseudocereal recognized for its nutritional composition and growing potential as a functional food ingredient. Beyond its high-quality proteins, dietary fiber, unsaturated lipids, and antioxidant compounds, increasing evidence suggests that the potential health-related effects of kiwicha may depend, at least partly, on the bioaccessibility of these components after gastrointestinal digestion rather than on their native composition alone. This narrative review examines how the structural organization of the kiwicha food matrix influences gastrointestinal digestion, nutrient bioaccessibility, and physiological functionality. It summarizes current evidence regarding protein, starch, lipid, and phenolic compound digestibility, emphasizing the release of bioactive peptides, antioxidant compounds, and other potentially bioactive metabolites during digestion. Furthermore, the review also discusses how processing technologies, including germination, extrusion, cooking, roasting, and fermentation, modify the food matrix, may increase nutrient bioaccessibility, and influence the functional performance of kiwicha-based foods. Critically, the available evidence indicates that processing-induced improvements in digestibility or bioaccessibility should not be interpreted directly as evidence of enhanced physiological functionality. Most studies remain based on in vitro digestion and biochemical assays, whereas evidence integrating intestinal absorption, systemic bioavailability, and human responses is scarce. Therefore, the main conclusion of this review is that the nutritional and potential functional relevance of kiwicha should be understood as a continuum linking food-matrix structure, processing-induced structural changes, gastrointestinal release, bioaccessibility, intestinal absorption, and ultimately physiological response.

## 1. Introduction

**Figure 1:**
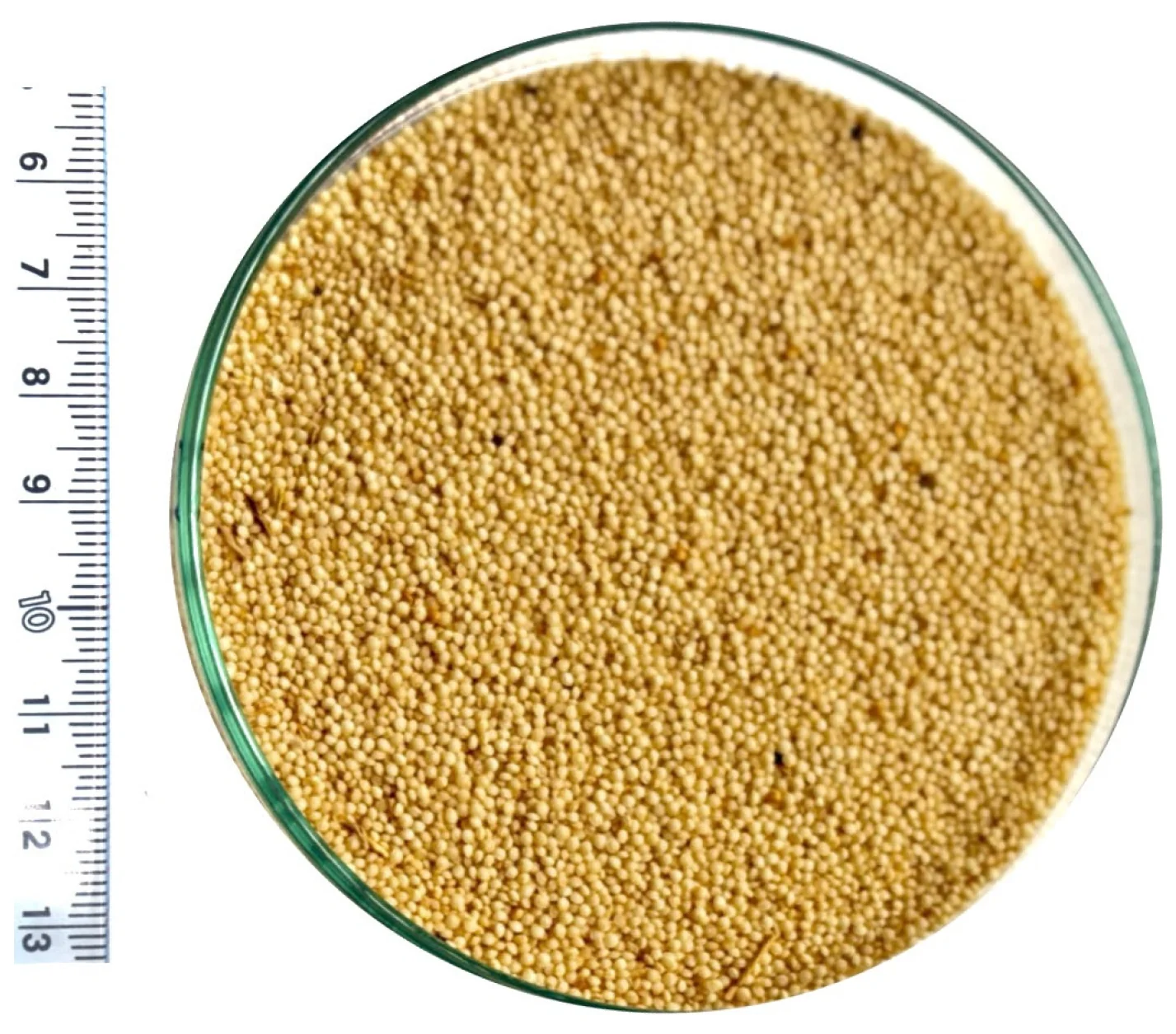
Surface appearance of the Centenario variety of kiwicha, with a scale in centimeters (cm).

The growing interest in healthier and more sustainable dietary patterns has increased scientific attention toward Andean grains and pseudocereals, among which kiwicha (*Amaranthus caudatus*) (Figure 1), has emerged as a promising ingredient because of its high nutritional density and diverse bioactive constituents [1,2,3]. Kiwicha typically contains 12–19% protein, with lysine levels double those of conventional cereals, together with dietary fiber, essential minerals, unsaturated lipids, squalene, and phenolic compounds [2,4,5]. However, these constituents’ nutritional and functional relevance cannot be inferred solely from their concentration in the raw grain. Their potential physiological significance ultimately depends on their release from the food matrix during gastrointestinal digestion, their resulting bioaccessibility, and, subsequently, their capacity to cross the intestinal barrier and become systemically bioavailable [6,7,8].

A recurring limitation in the scientific literature is that many studies report only the proximate composition and total phenolic content of raw kiwicha, without considering how gastrointestinal digestion affects the bioaccessibility of these nutrients and bioactive compounds and their subsequent availability for intestinal absorption [8,9,10]. This gap between the reported content and actual absorption highlights the critical need to assess the fate of nutrients during digestion. There is, therefore, a research gap regarding the relationship between the structural organization of the kiwicha matrix and its digestive behavior [8,11,12]. Structurally, kiwicha is recognized as a complex multicomponent matrix in which submicron-sized starch granules (0.5–2 µm) of a predominantly waxy nature are physically encapsulated within a dense network of storage proteins (albumins and globulins) and fibers rich in xyloglucans [8,13,14]. This microarchitecture acts as a natural physical barrier that influences the kinetics of hydrolysis and the release of nutrients [13,15].

With regard to their bioprocessing using technologies such as controlled germination and extrusion, these technologies can contribute to disrupting structural barriers, enabling the reduction of antinutrients such as phytic acid and potentially increasing the release of bioactive low-molecular-weight peptides (<3 kDa) with reported in vitro ACE-inhibitory and antioxidant activities [8,10,11,16]. The evaluation of these effects using standardized in vitro digestion models, such as the INFOGEST 2.0 protocol, provides valuable evidence for understanding how processing may influence nutrient digestibility, bioaccessibility, and starch hydrolysis in kiwicha-based foods [8,17,18].

The present review adopts an integrated digestion-centered framework that links food-matrix structure and processing-induced modifications with gastrointestinal digestion and bioaccessibility, and subsequently examines the available evidence on intestinal absorption and physiological functionality. This framework distinguishes between compounds released during digestion and those for which evidence of absorption or physiological effects is available, thereby highlighting the main translational gaps in the current literature.

Thus, the growing interest in kiwicha, together with the diversification of its processing methods and applications, highlights the need for a comprehensive consolidation of knowledge. In this context, the aim of this review is: (i) to analyze kiwicha as a food matrix, (ii) to describe its gastrointestinal digestion, (iii) to evaluate the bioaccessibility of nutrients and bioactive compounds, (iv) to examine the effect of processing on digestibility and bioaccessibility, and (v) to discuss its health implications, as well as future prospects, challenges, and research gaps.

## 2. Methodology

A narrative literature review was conducted to critically examine the current evidence on the structural characteristics, gastrointestinal digestion, nutrient and bioactive compound bioaccessibility, processing effects, and potential health-related functionality of kiwicha (*Amaranthus caudatus* L.). The structured search strategy was used to improve the transparency and coverage of the narrative review and was not intended to constitute a systematic or semi-systematic review. The literature search was performed in Scopus, Web of Science, and ScienceDirect, covering studies published from January 2021 to January 2026. Earlier studies were also considered when they provided relevant foundational information on the composition, structure, digestion, or functional properties of *A. caudatus*.

The search strategy combined the terms “*Amaranthus caudatus*”, “kiwicha”, and “amaranth” with keywords related to gastrointestinal digestion, nutritional quality, bioactive compounds, processing, and food applications. The main search terms included “gastrointestinal digestion”, “in vitro digestion”, “digestibility”, “protein digestibility”, “starch digestibility”, “bioaccessibility”, “bioavailability”, “bioactive peptides”, “phenolic compounds”, “antioxidant activity”, “nutritional composition”, “food matrix”, “functional properties”, “techno-functional properties”, “extrusion”, “germination”, “fermentation”, “cooking”, “roasting”, “thermal processing”, “food applications”, “bakery products”, “gluten-free products”, and “functional foods”. These terms were combined using the Boolean operators AND and OR and searched in the title, abstract, and keywords, where permitted by each database.

Original research articles and review articles published in English or Spanish were considered. Studies were included when they provided relevant information on at least one of the following topics: (i) structural and nutritional characteristics of kiwicha; (ii) gastrointestinal digestion and digestibility of its proteins, starch, lipids, or bioactive compounds; (iii) bioaccessibility or bioavailability of nutrients and phytochemicals; (iv) effects of processing technologies on food-matrix structure and digestive behavior; or (v) potential physiological functionality associated with digestion-derived compounds. Studies on other *Amaranthus* species or pseudocereals were considered only when they provided relevant comparative or mechanistic information that could not be obtained directly from *A. caudatus*. Duplicate records, conference abstracts, studies lacking sufficient methodological information, and publications not directly related to the scope of the review were excluded.

The identified publications were evaluated according to their relevance to the predefined thematic scope of this narrative review rather than through a formal systematic-review screening process. Particular attention was given to the type of experimental model used (in vitro digestion, cellular, animal, or human studies), processing conditions, analytical methods, and measured outcomes. Evidence from *A. caudatus* was distinguished from findings derived from other *Amaranthus* species or food matrices to avoid unsupported extrapolation. Furthermore, bioaccessibility was differentiated from bioavailability according to whether the studies assessed gastrointestinal release alone or included evidence of intestinal absorption or systemic availability.

## 3. Kiwicha as a Food Matrix

### 3.1. Food Matrix Concept

Structurally, kiwicha consists of a complex multicomponent matrix in which small starch granules are embedded in a dense network of storage proteins and dietary fibers, which confers techno-functional properties on it in baked goods and pasta products [8,13]. The results show that, when incorporated into dough formulations, this matrix increases the consistency, firmness, and cohesiveness of the system, altering the water absorption capacity and apparent viscosity compared with matrices based exclusively on conventional cereals [19].

This behavior is due to the nature of kiwicha starch, which is predominantly of the waxy type (low in amylose), as well as to the presence of fibers rich in pectic substances and xyloglycans, which create a steric hindrance within the viscoelastic network. During thermal or mechanical processing, such as extrusion, the proteins undergo denaturation which, combined with the gelatinization of the starch, allows the formation of highly viscous amorphous complexes where hydrogen bonds and disulfide bridges stabilize a new three-dimensional structure, limiting water migration and improving gas retention [19].

It is for this reason that, when comparing these results with previous research, there is a consensus that substituting pseudocereal flours increases the consistency index of the dough; however, kiwicha differs from other raw materials such as tarwi in that it has a moderate lipid content which acts as a lubricant, facilitating the flow of the dough and resulting in a softer crumb and finer porosity compared with the stiffer legume matrices [19,20,21]. Furthermore, these results suggest that the functionality of kiwicha may depend on the interactions between starch, proteins, lipids and fiber, rather than on the individual composition of each component, thereby supporting the concept of the food matrix. Consequently, technologies such as extrusion or germination can modulate these interactions to simultaneously modulate the technological quality, digestibility and functional value of the ingredient.

### 3.2. Structural Organization

Kiwicha has a complex multicomponent matrix in which polygonal starch granules are embedded within a dense network of storage proteins [13,22,23]. Electron micrographs show starch granules closely associated with protein bodies, forming irregular thin-lamellar structures or spherical and oblong aggregates measuring 10–30 µm [13,24,25].

The low amylose content of kiwicha starch, generally reported at approximately 5–7%, is associated with a predominance of amylopectin and may influence its interactions with surrounding proteins [23,25]. During kneading or extrusion processes, the protein fractions (dominated by albumins and 11S globulins) undergo unfolding and aggregation via hydrogen bonds, ionic interactions, hydrophobic interactions and disulfide bridges, forming a cross-linked network that restricts the free expansion and swelling of the starch granules [13,22,24].

This structural arrangement has direct implications for gastrointestinal digestion. Although the small starch granules provide a relatively high surface area for enzymatic interaction, their effective accessibility depends on the integrity of the surrounding protein network and other structural barriers. Protein aggregation may physically restrict *α*-amylase diffusion and starch swelling, whereas progressive proteolysis during gastrointestinal digestion can disrupt this barrier and increase starch accessibility. In parallel, fiber-rich cell walls may further restrict enzyme penetration and nutrient release, while the distribution of lipids within the matrix and their interactions with starch and proteins may modify substrate accessibility. However, direct evidence regarding lipid digestion and intestinal absorption in kiwicha remains limited.

All of this is highly significant for the food industry, given that the encapsulation of starch within the protein network may reduce its accessibility to digestive enzymes, potentially slowing starch hydrolysis and modulating the predicted glycemic response, while also contributing to improved viscoelastic stability in gluten-free products [8,26]. Furthermore, this structural robustness enables the matrix to withstand high shear and temperature conditions without losing its functional integrity [22,23].

When comparing these results with previous literature, it is evident that kiwicha has significantly smaller and more polygonal granules than the bimodal lenticular granules of wheat (~25 µm) [13]. However, unlike other species such as *A. cruentus*, kiwicha is characterized by greater amylopectin branching and superior stability under mechanical stress, which may contribute to its technological suitability for extrusion and bread-making applications [23,25]. Thus, digestion outcomes arise from the combined effects of starch granule characteristics, protein-network organization, fiber structure, and lipid distribution rather than from any single component in isolation. Table 1 summarizes these structure–mechanism–digestion relationships and highlights the current evidence for each matrix component. Consequently, optimizing the degree of matrix modification is a key factor in balancing technological performance with nutrient digestibility and bioaccessibility.

### 3.3. Natural Barriers to Digestion

Microstructural studies of kiwicha suggest that cell walls and the close association between starch granules and storage proteins can affect the rate of digestion [12,13,24]. Cell walls contain dietary fiber and other non-starch polysaccharides that may restrict enzyme access to nutrients within intact grain tissue [1,27,28]. Proteins surrounding the starch granules may further limit their swelling and accessibility to pancreatic α-amylase during intestinal digestion [8,13,23].

This structural organization is relevant to food formulation because it may influence the rate of starch digestion. Its effect on the glycemic response, however, depends on the composition and processing of the final product and requires direct evaluation [8,10,18].

These structural features may slow the initial enzymatic hydrolysis of kiwicha starch. A direct comparison with wheat, however, requires studies conducted under the same processing and digestion conditions [11,25]. Bioprocessing through germination or extrusion can contribute to disrupting these structural barriers through different mechanisms. Germination promotes the activity of endogenous hydrolytic enzymes, such as phytases and proteases, which contribute to the degradation of cell-wall and protein structures, whereas extrusion induces thermomechanical modifications of starch, proteins, and cell-wall components that may increase nutrient accessibility [1]. These structural barriers may be useful for developing foods with a slower rate of starch digestion. Processing could help regulate their effects, although any reduction in glycemic response must be confirmed in the final food product. In this context, the challenge lies in modulating the integrity of the matrix using processing technologies without compromising its functional properties.

## 4. Gastrointestinal Digestion of Kiwicha

**Figure 2:**
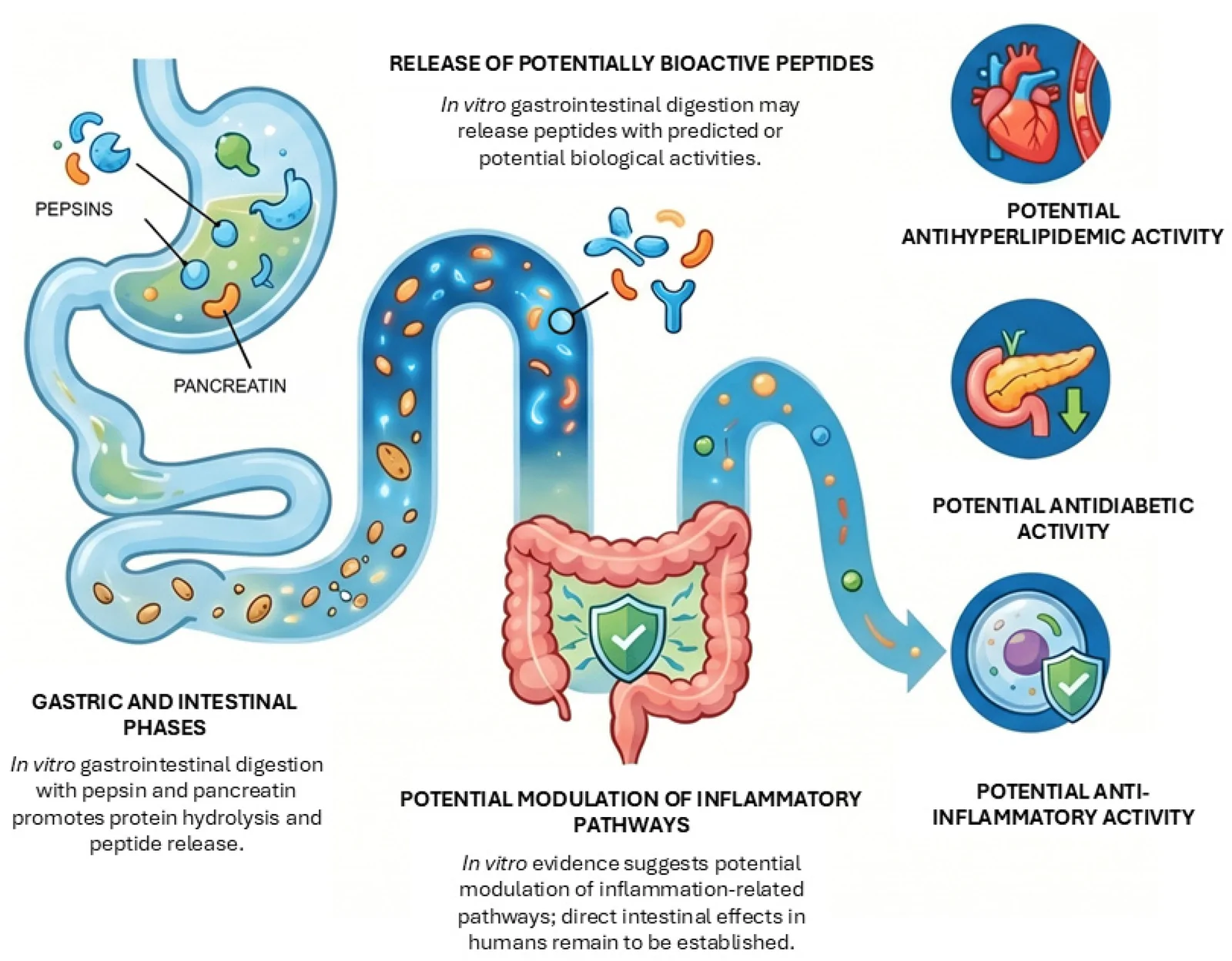
Gastrointestinal digestion of kiwicha proteins and release of potentially bioactive peptides. Biological activities shown represent potential or predicted effects primarily supported by in vitro evidence and should not be interpreted as evidence of intestinal absorption or systemic physiological effects in humans.

Simulated gastrointestinal digestion of kiwicha shows the release of compounds with potential biological activity and substantial protein hydrolysis (Figure 2). Germination and certain cooking methods may further increase protein digestibility, depending on the processing conditions [5,6,29]. The evaluation of kiwicha-based products under the standardized INFOGEST 2.0 protocol has shown the stability and bioaccessibility of certain nutrients and bioactive compounds under in vitro gastrointestinal conditions, with in vitro protein digestibility of up to 79.18%, while cooked products enriched with sprouted grains manage to retain significant levels of bioaccessible γ-aminobutyric acid (GABA) and phenolic compounds by the end of the intestinal phase [8,10,18,29]. These findings represent in vitro evidence of digestive release and bioaccessibility and should not be interpreted as direct evidence of intestinal absorption or systemic physiological effects.

### 4.1. Oral Phase

During the oral phase, commonly simulated for 2 min, kiwicha-based matrices are exposed to hydration and salivary α-amylase, initiating starch hydrolysis under simulated digestion conditions [8,18]. These initial changes may influence how the food matrix is digested in the subsequent gastric and intestinal phases [8,18]. In studies using processed kiwicha-based matrices, such as bread or pasta, the formation of the food bolus in a 1:1 ratio with salivary fluid has been associated with rapid initial hydrolysis within the first 5 min of the digestive process and the release of soluble compounds [10,18].

This behavior may be partly associated with the unique morphology of the small kiwicha starch granules, which may provide a relatively large surface area for interaction with salivary α-amylase [8,11]. During this brief period, water molecules may penetrate the starch matrix, promoting granule swelling and structural disruption, particularly in previously bioprocessed or cooked grains [11,15]. However, the extent of this initial change may be modulated by the grain’s high phenolic content, as these compounds may interact with *α*-amylase and potentially reduce its activity, thereby slowing starch hydrolysis [16,18]. These mechanistic interpretations are primarily based on established behavior in starch- and phenolic-rich food matrices. Although they provide plausible explanations for the observed digestive behavior of kiwicha, direct evidence demonstrating the magnitude and specific contribution of hydration, granule swelling, and phenolic–α-amylase interactions during the oral digestion of *A. caudatus* remains limited.

The scientific significance of these initial changes lies in the fact that the breakdown of the matrix in the mouth may influence the kinetics of gastric and intestinal digestion; efficient hydration and partial degradation of the starch may modify bolus properties and influence the subsequent action of pepsin and pancreatic proteases [8].

From a nutritional perspective, these characteristics may be relevant to the development of foods with modulated starch digestibility, although their effect on glycemic response depends on the composition and processing of the final product. Furthermore, differences in hydration kinetics between kiwicha and wheat have been reported and may be partly associated with differences in starch granule size and morphology [8]. Similarly, while other grains such as quinoa exhibit a more compact amylopectin structure that restricts amylolysis during the oral phase, kiwicha displays relatively rapid initial starch hydrolysis, reaching approximately 15–27% in suspension-based experiments [8,15,30]. These findings suggest that the structural characteristics of kiwicha may influence early starch hydrolysis; however, direct comparisons among pseudocereals should be interpreted cautiously because processing conditions, food matrices, and digestion protocols may differ among studies.

Taken together, these results suggest that the oral phase contributes to the subsequent digestive behavior of kiwicha, as the interactions between its granular structure other matrix componentsmay influence the initial rate of starch hydrolysis. Consequently, controlling the pre-processing of the grain could represent a potential strategy for modulating starch digestibility and the subsequent digestive behavior of kiwicha-based foods.

### 4.2. Gastric Phase

During the simulated gastric phase of kiwicha digestion, a breakdown of the food matrix is observed, characterized by extensive protein denaturation and the onset of enzymatic hydrolysis, increasing the bioaccessibility of total phenolic compounds, reaching values of up to 362.96 mg GAE·100 g^−1^ DW in enriched matrices [18]. However, the mechanisms underlying these changes have not been fully characterized specifically in *A. caudatus*. Based on established principles of gastric digestion and evidence from comparable food matrices, the acidic gastric environment (pH 1.2–3.0) may promote the unfolding of protein tertiary structures through the disruption of non-covalent interactions, including hydrogen bonds and hydrophobic interactions [24,31]. This structural modification may increase the accessibility of peptide bonds to pepsin and facilitate protein hydrolysis and the release of peptides and matrix-associated compounds [8,29,31]. Therefore, these mechanisms should be interpreted as plausible explanations for the observed digestive changes rather than as fully demonstrated kiwicha-specific pathways.

The implications of these results are relevant to understanding the digestive behavior of kiwicha, as changes occurring during the gastric phase may influence subsequent intestinal digestion. Effective gastric protein denaturation may facilitate the subsequent release of low-molecular-weight peptides (<3 kDa) with potential antihypertensive and anti-inflammatory activities during the intestinal phase [31]. Furthermore, the observed stability of phytic acid during this stage suggests mineral accessibility may be influenced more by prior bioprocessing, such as germination, than by gastric acid degradation alone [8,18].

When comparing these findings with previous research, kiwicha exhibits an initial in vitro protein digestibility (77.9%) higher than that reported for other pseudocereals such as buckwheat and legumes such as pigeon peas under identical gastric conditions [29]. While studies indicate that quinoa globulins are also highly susceptible to pepsin, a relatively rapid initial hydrolysis of kiwicha proteins has also been reported [24,29]. However, direct comparisons among kiwicha, quinoa, conventional cereals, and other protein sources should be interpreted cautiously because differences in protein composition, food matrix, and experimental conditions may influence digestion kinetics.

Taken together, these results suggest that the efficiency of the gastric phase depends not only on the protein composition of kiwicha, but also on the organization of its food matrix, which may influence the release of peptides and bioactive compounds. In this regard, the controlled modification of this matrix using processing technologies may represent a potential strategy to enhance the bioaccessibility of functional compounds without compromising their stability during digestion.

### 4.3. Intestinal Phase

The intestinal phase of simulated kiwicha digestion represents a major stage of extensive hydrolysis, during which in vitro protein digestibility reaches values of up to 79.18 per cent and the bioaccessibility of total phenolic compounds increases significantly, reaching levels of between 357–638 mg GAE·100 g^−1^ DW by the end of this process [8,29]. During this phase, small peptides and soluble nutrients are released from the grain matrix. This release is explained by the synergistic action of pancreatin (a mixture of trypsin, chymotrypsin, elastase and carboxypeptidases) and bile salts in a neutral pH environment (~7.0), which catalyses the breakdown of the multicomponent matrix [8,31].

The breakdown of storage proteins not only releases essential amino acids but also liberates antioxidants and polyphenols bound to the cell wall, while the small size of the starch granules allows pancreatic amylases to access them, despite the initial steric hindrance posed by dietary fiber [8,15].

The implications of these findings are relevant to understanding the potential postdigestion functionality of kiwicha. The persistence of bioactive peptides and polyphenols after intestinal digestion provides evidence of their bioaccessibility under the evaluated in vitro conditions but does not necessarily imply intestinal absorption or systemic bioavailability. Their reported ACE-inhibitory and glucose-modulating activities are primarily indicative of in vitro bioactivity and should not be interpreted as evidence of antihypertensive, antidiabetic, or other cardiometabolic effects in vivo. Such physiological relevance requires evidence of intestinal transport and systemic bioavailability, followed by validation in animal models and, ultimately, human studies [8,32,33].

The available findings suggest that intestinal starch hydrolysis in kiwicha depends on both grain characteristics and prior processing [8]. In the study reporting 67.4% starch hydrolysis after boiling, roasting was associated with slower digestion. These results indicate that processing can alter starch accessibility, but they do not establish a lower GI than wheat. That comparison requires testing the final foods under equivalent conditions [15].

Taken together, these results suggest that the digestive response to kiwicha is influenced by the interaction between its structural components and the prior processing of the grain, supporting the concept of the food matrix. Consequently, modulating this matrix using processing technologies may represent a useful strategy for developing foods with improved digestibility and bioaccessibility of bioactive compounds and with modulated starch digestion, these digestive outcomes should not be extrapolated directly to systemic cardiometabolic benefits, and effects on glycemic response require confirmation in the final food product and, ultimately, in human studies.

## 5. Bioaccessibility of Nutrients and Bioactive Compounds

### 5.1. Protein Digestibility and Peptide Release

In vitro gastrointestinal digestion of kiwicha protein isolates and enzymatically produced hydrolysates has shown protein digestibility values of 77.97–79.18% and the generation of low-molecular-weight peptide fractions under the evaluated experimental conditions [29,34]. These findings refer specifically to isolated or hydrolyzed protein systems and should not be extrapolated directly to intact kiwicha grains or finished food products [29,34]. Using standardized protocols such as INFOGEST 2.0, it has been observed that these peptide fractions exhibit greater stability under gastrointestinal conditions and account for a large proportion of the biological activity detected following the gastric and intestinal phases [10,31].

From a biochemical perspective, this high digestive efficiency is explained by the composition of kiwicha’s reserve fractions, which are dominated by albumins (~40%) and globulins (~20%)—fractions that are more susceptible to hydrolysis by pepsin and pancreatin than the prolamins found in conventional cereals [29,35,36]. During the digestive process, enzymes break down protein-starch complexes and cell wall barriers, allowing proteases to cleave specific peptide bonds and release ‘encrypted’ sequences that have an affinity for key biological receptors [5,31,36]. However, the extent of this hydrolysis can be modulated by technological factors such as heat treatment or extrusion, which can induce protein aggregation or Maillard reactions, thereby reducing enzymatic accessibility.

The peptides released during digestion are of interest because some may retain biological activity after gastrointestinal hydrolysis. Various studies have attributed antioxidant properties, ACE inhibitory activity and the ability to modulate inflammatory pathways associated with the NLRP3 inflammasome to the fractions released during digestion; however, these findings primarily reflect peptide bioactivity demonstrated under experimental conditions and should not be interpreted as evidence of the prevention of cardiometabolic or chronic inflammatory disorders in humans [29,35,37]. Nevertheless, its amino acid profile, with lysine levels double those of wheat, supports the nutritional relevance of kiwicha protein for specialized diets and gluten-free products [5,35,38]. That said, the actual bioavailability of these amino acids may depend on effective digestion and possible interactions with other dietary components.

The nutritional and digestive characteristics of kiwicha proteins should be interpreted according to the specific metric and experimental system used. In vitro protein digestibility, degree of hydrolysis, amino-acid composition, PDCAAS, and DIAAS represent distinct aspects of protein quality and should not be directly compared. Likewise, peptide bioactivity reflects specific biological activities measured in biochemical or cellular assays rather than overall protein quality. Although kiwicha contains a nutritionally relevant proportion of sulfur-containing amino acids and can generate peptide fractions during gastrointestinal digestion, these characteristics should be interpreted independently from standardized protein-quality indices such as PDCAAS and DIAAS [29,35,39].

Importantly, the evidence supporting these digestive and biological outcomes is derived from different experimental levels and should therefore not be interpreted as having equivalent physiological relevance. As summarized in Table 2, evidence on protein digestibility and peptide release is predominantly derived from in vitro gastrointestinal digestion and acellular assays, whereas evidence from cellular and animal models is more limited and varies according to the evaluated outcome. Most importantly, direct human evidence remains scarce or unavailable for several of these effects. This distinction is essential because bioaccessibility and in vitro biological activity do not necessarily imply intestinal absorption, systemic bioavailability, or physiological efficacy in humans.

Nevertheless, this comparison should be interpreted with caution, as the assessment methods (PDCAAS vs. DIAAS—digestible indispensable amino acid score) and experimental conditions may vary significantly between studies, affecting the direct comparability of the results.

Therefore, future studies should integrate standardized INFOGEST digestion models with advanced intestinal absorption systems, in vivo models, and human clinical trials to determine whether the bioactivities observed in vitro translate into measurable physiological effects under real-world consumption conditions.

### 5.2. Starch Digestibility

The digestion of starch in products made from kiwicha may vary depending on processing and food-matrix structure, with an estimated GI of approximately 83.16 reported in enriched breads and reductions in the hydrolysis index (HI) following optimized germination and baking processes [10,18]. However, these values should be interpreted with caution, as the estimated GI can vary considerably depending on the food formulation, matrix structure, and in vivo testing conditions.

Although kiwicha contains a waxy starch (amylose < 5–7%), it is typically associated with slower digestion kinetics than wheat, characterized by a lower proportion of rapidly digestible starch (RDS) and greater stability during intestinal transit [14,23,25,35]. This behavior is particularly interesting, as it contradicts the classic relationship between low amylose content and high digestibility. Consequently, it suggests that structural and matrix factors play a more decisive role than the composition of the starch per se.

Various studies attribute this phenomenon to the characteristic microarchitecture of the kiwicha matrix, where extremely small granules remain embedded in a compact network of storage proteins and dietary fiber [13,15,25,35]. In fact, this organization creates a steric hindrance that limits the expansion of the granules and restricts the access of pancreatic *α*-amylase to the catalytic sites, slowing down the conversion of complex polymers into free glucose [8,10,15,25]. Furthermore, the presence of phenolic compounds in the matrix may contribute to the inhibition of hydrolytic enzymes under in vitro conditions [10,16,18]. However, the extent of this inhibitory effect depends on the bioaccessibility of phenolic compounds following processing and digestion, and its physiological relevance remains to be established.

These characteristics support the potential use of kiwicha as an ingredient in food formulations designed to modulate in vitro starch digestibility [2,8,10,37]. However, the available evidence does not establish that kiwicha consumption improves glycemic control or metabolic health in humans, and such effects require confirmation through appropriately designed in vivo and clinical studies.

Furthermore, a lower estimated GI has been reported for specific kiwicha-containing formulations compared with the corresponding wheat controls [10,18,25]. However, previous research highlights that the processing method is a determining factor: while boiling increases the percentage of hydrolysis to up to 67.4%, dry heat processes such as roasting or popping promote slower in vitro starch digestion [8,15,22,40]. Therefore, optimizing processing technologies is a key strategy for modulating in vitro starch digestibility. However, estimated GI values and in vitro starch hydrolysis should not be interpreted as equivalent to an in vivo glycemic response.

Overall, although the results obtained using in vitro models are promising, further studies are required that integrate standardized gastrointestinal digestion, advanced intestinal absorption systems, in vivo evaluations, and clinical trials in humans to determine whether the differences in starch digestibility and estimated GI observed experimentally translate into measurable effects on postprandial glycemic responses and metabolic outcomes in humans.

### 5.3. Lipid Composition and Current Evidence on Digestive Bioaccessibility

Kiwicha contains a lipid fraction accounting for between 5.1–9.5% of the grain, characterized by a high proportion of unsaturated fatty acids, with linoleic acid accounting for approximately half of the total profile [10,41,42]. The results show that, despite the reduction in total fat content during bioprocesses such as germination (which decreases from 7.10 to 4.60% due to metabolic consumption), the remaining fraction retains lipid compounds of nutritional interest, such as squalene and *β*-sitosterol [5,14]. However, this quantitative reduction could affect the ingredient’s energy density, which must be considered in specific applications.

The mobilization of lipids may be influenced by the structural organization of the grain. The lipid bodies located in the embryo and endosperm could become more accesible following hydration and disruption of the food matrix [1,5]. At the same time, the breakdown of the multicomponent matrix, where proteins and fibers act as physical barriers, could theoretically facilitate the access of pancreatic lipases to triglycerides. However, these mechanisms have not been quantitatively established for kiwicha, and direct evidence on the extent of gastrointestinal lipolysis remains limited [1,8]. However, the efficiency of this process may be influenced by the degree of processing, as intense heat treatments can induce lipid–protein interactions or oxidative processes may affect the accessibility of fatty acids.

Another important aspect is the high proportion of polyunsaturated fatty acids present in kiwicha, a characteristic that contributes to its nutritional value but also increases its susceptibility to oxidative processes during processing and storage [43]. Consequently, oxidative stability emerges as a critical factor in its industrial application.

From a compositional perspective, the presence of linoleic acid (>40%), oleic acid (>25%), phytosterols and squalene supports the nutritional interest of the kiwicha lipid fraction [34,38,43]. However, their presence alone does not demonstrate gastrointestinal bioaccessibility, intestinal absorption, or cardiometabolic effects. The physiological relevance of these compounds depends on their release during digestion, lipolysis, micellarization, and subsequent intestinal absorption, for which direct evidence in kiwicha remains limited. Kiwicha may therefore be a useful ingredient for foods that provide unsaturated lipids alongside other nutrients, although its effects on cholesterol regulation and lipid metabolism require further investigation [7,14].

In the varieties examined, kiwicha had a higher proportion of linoleic acid than the Mexican *A. hypochondriacus* varieties used for comparison, which had a higher proportion of palmitic acid. This difference should be interpreted in relation to the specific varieties and growing conditions studied [43]. Although kiwicha has a nutritionally relevant lipid profile, direct evidence demonstrating favorable lipid bioavailability remains insufficient. Excessive extrusion temperatures (>180 °C) may compromise the integrity of the unsaturated fatty acids [22,42,44]. Consequently, optimizing processing conditions is essential to preserve the stability and nutritional quality of the lipid fraction. Importantly, compositional differences among kiwicha, other amaranth species, and other pseudocereals should not be interpreted as evidence of differences in lipid release, gastrointestinal bioaccessibility, or intestinal uptake because direct comparative digestion studies are currently lacking.

Despite these advances, a significant knowledge gap remains regarding the digestive behavior of the lipid fraction of kiwicha. Most available evidence is compositional and focuses on total lipid content, fatty-acid profiles, phytosterols, and squalene rather than on gastrointestinal lipid digestion. Direct quantitative evidence on pancreatic lipolysis, incorporation of fatty acids and other lipid-soluble compounds into mixed micelles, gastrointestinal bioaccessibility, and intestinal uptake remains largely lacking. Therefore, the current evidence does not support conclusions regarding superior lipid release or bioavailability of kiwicha compared with conventional cereals or other pseudocereals. Further research is required that integrates dynamic models of digestion, micellization assays, intestinal absorption systems and in vivo studies to gain a more precise understanding of the bioavailability and physiological effects of its lipid compounds.

### 5.4. Phenolic Compounds and Antioxidant Bioaccessibility

Technological processing and gastrointestinal digestion of kiwicha have a dual effect on phenolic compounds, whereby bioprocesses such as germination and moderate extrusion significantly enhance their release, while intense heat treatments induce their progressive degradation [16,22]. Controlled germination for 72 h can increase the total phenolic compound (TPC) to as much as 926 mg GAE·100 g^−1^ DW in black ecotypes [5,8]. Complementarily, standardized digestion models based on INFOGEST 2.0 show that the intestinal phase is a critical stage for the release of these molecules, with bioaccessibility levels exceeding those observed in unprocessed grain [8,10,18]. However, the extent of these increases varies considerably depending on the genotype, processing conditions and the nature of the food matrix, which makes direct comparison between studies difficult.

The mechanisms responsible for this behavior are linked to both structural changes and responses induced during processing. The activation of endogenous enzymes (glucanases and esterases) that degrade the cell wall and protein-starch complexes, releasing bound forms into their free state, coupled with de novo synthesis via the phenylpropanoid pathway during shoot growth [1,5,8,10]. Conversely, the degradation observed at temperatures above 180–190 °C or during prolonged cooking is due to thermal oxidation and the degradation of aromatic structures [16,22]. However, it should also be borne in mind that certain thermal processes can generate derivative compounds (e.g., Maillard products with antioxidant activity), which complicates the direct interpretation of functional loss.

This evidence has significant implications for the development of functional foods, as it supports the use of bioprocessing strategies to enhance the release and bioaccessibility of phenolic compounds in kiwicha-based foods. However, whether these increases translate into systemic antioxidant effects or clinically relevant health benefits remains to be established [8,18,29].

Compared with other cereals, kiwicha has a higher potential for the release of phenolic compounds than wheat and other conventional cereals, where the refining process removes the bran layers in which these compounds are concentrated [8,18]. Furthermore, although pseudocereals such as quinoa and buckwheat also show increases in bioaccessibility during digestion, kiwicha stands out for its accumulation of specific compounds such as caffeoylquinic acid and rutin, which are associated with good gastrointestinal stability [1,10]. However, this advantage may vary depending on genotype and growing conditions; consequently, the standardization of raw materials emerges as a key challenge for its consistent industrial application [45]. A summary of the main findings on the bioaccessibility of nutrients and bioactive compounds in kiwicha is presented in Table 3.

## 6. Effect of Processing on Digestibility and Bioaccessibility

Technological processing of kiwicha is a key factor in modulating the structural organization of its food matrix and influencing the digestibility and release of its essential nutrients and bioactive compounds [22,44,48]. Technologies such as germination, extrusion and thermal cooking (Table 4) can reduce the presence of antinutrients, specifically phytic acid, tannins and protease inhibitors, which may limit mineral availability and protein digestion [1,33,48,49]. Furthermore, it has been reported that processes such as extrusion and popping induce protein denaturation and starch gelatinization, which can modify the accessibility of these macronutrients to digestive enzymes, although the magnitude of these effects depends on the processing conditions and food matrix [22,40,44,50].

Complementarily, biotechnological strategies such as germination and fermentation activate endogenous enzymes (phytases, glucanases and proteases) that break down protein-starch complexes and fiber-rich cell walls, releasing bioactive low-molecular-weight peptides and increasing the extractability or release of phenolic compounds and GABA [5,10,18,51]. Consequently, the integration of these treatments may contribute to improving the digestibility and in vitro digestibility and accessibility of kiwicha nutrients and bioactive compounds, supporting its potential application in functional foods [15,22].

**Table 4:** Quantitative effects of processing methods on structural characteristics, in vitro digestion parameters, and bioactive compounds of *Amaranthus caudatus* L.

ProcessingMethod	Target Component	Processing Conditions	Structural/Physicochemical Changes	Digestion Model/Evaluation Method	Quantitative Result	Evidence Level	References
Germination	Protein	20–26 °C for 18–48 h (or malting at 22–30 °C for 24–48 h)	Endogenous proteolysis of storage proteins (albumins, globulins, and glutelins) and modification of the protein–starch matrix	In vitroprotein digestion (pepsin–pancreatin)/Bradford assay	In vitroprotein digestibility varied according to germination/malting conditions, ranging from 75.0 to 83.6%; crude protein content changed by +5% to +54%, while proline increased by up to 500% after 24 h	In vitrodigestion/biochemical	[1,5,18,29]
	Starch	20–26 °C for 24–48 h	Activation of endogenous α- and β-amylases, partial alteration of starch-granule surfaces, and changes in amylopectin molecular structure	Enzymatic hydrolysis assay/In vitrostarch-fraction determination	Total starch and rapidly digestible starch (RDS) decreased, whereas resistant starch (RS) remained comparatively stable; changes in the amylose/amylopectin ratio were also reported	Acellular/biochemical	[1,22,44]
	Phenolic compounds	20–26 °C for 24–72 h	Enzymatic release of bound phenolics and increased accumulation of flavonoids, including rutin and isoquercitrin, and betalains	Folin–Ciocalteu assay (TPC)/HPLC-PDA/ABTS, DPPH, and FRAP assays	TPC increased by up to 212.98% after 24 h; increased concentrations of rutin and isoquercitrin were also reported	Acellular/biochemical	[1,33,44]
Extrusion	Protein	Twin-screw extrusion; 100–158 °C, 365 rpm or 40–52 Hz, and 11–20% feed moisture	Thermal denaturation and unfolding of proteins, with protein–protein and protein–starch interactions induced by heat and shear	In vitro protein digestibility ranged from approximately 75.0 to 82.6%, depending on extrusion conditions; amino-acid composition was comparatively preserved under the evaluated conditions	In vitro protein digestibility ranged from approximately 75.0 to 82.6%, depending on extrusion conditions; amino-acid composition was comparatively preserved under the evaluated conditions	In vitro digestion/biochemical	[22,40]
	Starch	Twin-screw extrusion; 100–158 °C, 13–20% moisture, 365 rpm	Disruption of native starch granules, gelatinization, partial molecular degradation, and increased water solubility	In vitro starch hydrolysis (α-amylase + amyloglucosidase)/RVA pasting analysis	Increased starch hydrolysis and digestible starch fractions were reported, together with reductions in RS and slowly digestible starch (SDS); predicted glycemic index (pGI) increased under some extrusion conditions	In vitro digestion/biochemical	[22]
	Lipids	Twin-screw extrusion; 100–158 °C, 11–16% moisture	Disruption of lipid-containing structures and formation of starch–lipid and protein–lipid interactions	Solvent extraction/lipid-content and composition analyzes	Extractable lipid fraction decreased by approximately 0.40–2.9%, whereas reported total lipid contents ranged from 7.0 to 8.17 g/100 g DW under the evaluated conditions	Acellular/compositional	[22]
Cooking (boiling)	Starch	Boiling at 100 °C for 10–30 min; final moisture approximately 64.82 ± 0.31%	Hydration and swelling of starch granules, loss of native crystalline organization, and extensive gelatinization	Simulated in vitro GI digestion/α-amylase hydrolysis/first-order kinetic modeling	Significant increase: hydrolysis reaching ~67.4%	In vitro digestion	[15,22]
	Phenolic Compounds	Boiling at 100 °C for 15–30 min	Thermal modification of the food matrix and release and/or leaching of water-soluble phenolic compounds	Folin–Ciocalteu assay (TPC)/simulated GI digestion/FRAP and ABTS assays	TPC in boiled grain was 470.21 mg GAE/100 g DW; after 360 min of simulated GI digestion, released TPC reached 143.93 mg GAE/100 g DW in intact grain and 281.02 mg GAE/100 g DW in slurry; FRAP reached 449.10 μmol FeSO_4_/100 g DW	In vitro digestion/acellular	[15,16]
Roasting/popping	Starch	Dry roasting at 150–200 °C for 15–60 s or popping at 200 °C for 15 s; final moisture approximately 8.17 ± 0.24%	Limited gelatinization under low-moisture conditions, rapid structural expansion during popping, and thermally induced matrix modifications	Simulatedin vitroGI digestion/*α*-amylase hydrolysis/first-order kinetic modeling	C∞ was 15.50 ± 0.97% for intact roasted grain (k = 0.0017 min^−1^) and 68.87 ± 1.04% for roasted slurry (k = 0.0171 min^−1^); RS was reduced to 0.05 ± 0.00%	In vitro digestion	[15,17,52]
	Protein	Dry roasting/popping at 150–200 °C for 15–60 s	Thermal protein denaturation and aggregation, with possible protein–carbohydrate interactions under intense heating	In vitro protein digestion/amino-acid analysis	Crude protein increased from 10.30 to 11.81 g/100 g DW in the Centenario variety; reported protein-digestion-related values and available lysine decreased under intense thermal processing, with available lysine reductions of up to 83%	In vitro digestion/biochemical	[14,34]
Fermentation	Protein	LAB or natural fermentation at 30–37 °C for 24–48 h	Microbial proteolysis of storage proteins, generation of lower-molecular-weight peptides and free amino acids, and reduction of some antinutritional compounds	In vitro digestion/peptide profiling/in vitro ACE-inhibition assay	Crude protein content increased by approximately 3% under the reported conditions; reductions in phytic acid and tannins and the release of peptides exhibiting in vitro ACE-inhibitory activity were reported	In vitro digestion/acellular	[8,34,36,44]
	Phenolic compounds	LAB fermentation at 30–37 °C for 24–48 h	Microbial enzymatic modification of phenolic conjugates, potentially increasing the proportion of free or extractable phenolic compounds	Folin–Ciocalteu assay (TPC)/DPPH, ABTS, and ORAC assays	Increased TPC and radical-scavenging capacity were reported in fermented matrices, together with changes in individual phenolic compounds, including caffeoylisocitric acid and rutin	Acellular/biochemical	[10,33,36,51]

**Note:** ABTS, 2,2′-azino-bis(3-ethylbenzothiazoline-6-sulfonic acid); ACE, angiotensin-converting enzyme; DPPH, 2,2-diphenyl-1-picrylhydrazyl; DW, dry weight; FRAP, ferric reducing antioxidant power; GAE, gallic acid equivalents; GI, gastrointestinal; LAB, lactic acid bacteria; ORAC, oxygen radical absorbance capacity; pGI, predicted glycemic index; RDS, rapidly digestible starch; RS, resistant starch; SDS, slowly digestible starch; TPC, total phenolic content. Evidence classified as in vitro digestion when simulated gastrointestinal or enzymatic digestion was performed and as acellular/biochemical when conclusions were based on compositional, enzymatic, or chemical assays without evidence of intestinal absorption or in vivo efficacy. Reported changes refer to the specific processing conditions and analytical methods of the cited studies and should not be interpreted as evidence of systemic bioavailability or health effects in humans.

### 6.1. Germination

The bioprocessing of kiwicha through controlled germination has proven to be an effective technology for modifying macronutrient digestibility and increasing the release or extractability of bioactive compounds, demonstrating significant increases in GABA content and in the total phenolic compound content, which can reach maximum values of 926 mg GAE·100 g^−1^ DW, alongside a marked reduction in antinutritional factors such as phytic acid [5,10].

These effects are closely linked to the metabolic reactivation of the seed during imbibition and the initial growth of the shoot. The activation of endogenous hydrolytic enzymes, including phytases, proteases and other degradative enzymes, promotes the partial breakdown of the protein–starch matrix and cell walls, facilitating the release of previously chelated minerals and phenolic compounds bound to the matrix [1,5]. At the same time, the synergistic action of these enzymes breaks down the physical protein–starch matrix and degrades the cell walls, thereby releasing previously chelated minerals and converting bound forms of phenols into their free state, while the specific activation of the enzyme glutamate decarboxylase efficiently catalyses the hyperaccumulation of GABA [5,10].

The implications of these findings may be relevant to the functional foods and nutrition industry, as they support the potential use of sprouted kiwicha flours as strategic ingredients in the design of products with enhanced nutrient bioaccessibility, such as infant formula, gluten-free breads and biscuits [5,10].

Compared with other processing methods, germination has been reported to modify and, under specific conditions, improve the nutritional and bioactive profile of kiwicha to a degree comparable to or greater than that of other Andean grains under similar conditions; it has also been reported that this bioprocess may improve the release and extractability of phenolic compounds and measured antioxidant capacity compared with severe thermal methods (such as popping or high-temperature extrusion), which can lead to the degradation of heat-sensitive phytochemicals and amino acids [5,10,14]. However, the extent of these improvements depends on variables such as ecotype, germination time, temperature and cultivation conditions, aspects that require greater standardization to facilitate comparison between studies and optimize their industrial application.

### 6.2. Extrusion

It is a high-temperature short-time (HTST) technology that can modify the in vitro digestibility of proteins and starch, while influencing the release and extractability of its bioactive components [22]. Available studies indicate that, under moderate processing conditions (~160 °C), an increase of up to 71.88% in antioxidant capacity and 20.32% in total phenolic compound content is possible due to the release of phytochemicals bound to the food matrix; however, the use of extreme temperatures (>180–190 °C) leads to irreversible oxidation and thermal degradation of these polyphenols [16,22].

The changes observed during extrusion result from the combined action of thermal energy, pressure, and shear forces. These conditions promote protein denaturation, starch gelatinization, and the partial breakdown of cell walls and other components of the food matrix [22]. This drastic structural reorganization reduces some of the physical barriers within the grain matrix, increasing the accessibility of proteins and starch to digestive enzymes such as proteases and pancreatic *α*-amylase [22].

Consequently, as with other Andean grains, extrusion under controlled parameters may represent a promising industrial strategy for developing gluten-free functional foods (such as snacks and cereals) that can modify macronutrient digestibility and promote the release of peptides exhibiting in vitro ACE-inhibitory activity [22,35,53]. The effects of extrusion depend on temperature, moisture content, screw speed, and residence time. Moderate conditions may improve nutrient accessibility, whereas more intense processing can degrade compounds sensitive to heat. Adjusting these variables is therefore necessary to improve digestibility while preserving the nutritional and functional properties of kiwicha.

### 6.3. Cooking

Thermal processing through cooking results in an increase in the in vitro digestibility of its macronutrients, achieving starch hydrolysis of up to 67.41% in the intestinal phase, alongside a variable pattern in the release of phenolic compounds, which may increase due to the breakdown of the matrix or decrease significantly due to leaching effects [15,54]. Physicochemically, this behavior is explained by high hydration during heating; water molecules penetrate into the starch granules, causing them to swell, disrupting their crystalline order, and leading to complete gelatinization [15]. This loss of native structure increases the accessibility of starch to pancreatic α-amylase and facilitates enzymatic hydrolysis. As for bioactive compounds, heat hydrolyzes the bonds that keep phenolic acids bound to the carbohydrate and protein matrix, releasing them into their free form; however, because they are water-soluble, these antioxidants rapidly migrate into the cooking water, resulting in substantial losses if the liquid is discarded [41,54].

From a technological and nutritional perspective, these findings suggest that boiling is an effective method for preparing foods with a smooth texture and increased starch hydrolysis under in vitro conditions, although retaining the cooking water (for example, in soups or beverages) may contribute to retaining phenolic compounds leached from the grain during cooking [41,54].

### 6.4. Roasting

Roasting can alter the digestion of kiwicha components in different ways. Under the conditions studied, it was associated with reduced starch hydrolysis and increased release of phenolic compounds during simulated digestion, along with higher measured antioxidant activity [15,27].

The limited water available during roasting may restrict starch gelatinization, while heating can promote Maillard reactions. Together with changes in the grain structure, these effects may reduce the accessibility of starch to digestive enzymes such as *α*-amylase. The contribution of each mechanism depends on the roasting conditions and remains to be established [15,27]. At the same time, heat treatment destabilizes the food matrix and breaks the covalent bonds that hold phenolic acids bound to macromolecules (such as cell wall fibers and proteins), releasing them into free or more extractable forms [27,54]. From a nutritional and technological perspective, these findings suggest that roasting may help modulate starch digestion and increase the release of compounds with antioxidant activity. Its effects on glycemic response and antioxidant availability in the body require evaluation in the final food product and in human studies [27,54].

However, the effects of roasting depend heavily on variables such as temperature, exposure time, and the moisture content of the grain. Excessively harsh conditions can compromise the stability of heat-sensitive compounds and reduce some of the observed benefits. Therefore, optimizing processing parameters is essential to balance starch digestibility, nutrient retention, and the availability of bioactive compounds. Overall, the available evidence suggests that roasting may be useful for modulating starch digestion and the extractability of bioactive compounds; however, claims regarding a low glycemic response or systemic antioxidant effects require confirmation through in vivo and human studies.

### 6.5. Fermentation

Fermentation may improve macronutrient digestibility and the availability of minerals such as zinc, calcium, and iron, although the magnitude of these effects depends on the microorganisms and processing conditions. Microbial activity can break down components of the food matrix and lower its pH, favoring the action of phytases. The resulting reduction in phytic acid may increase mineral solubility, while changes to the protein matrix may improve access for digestive enzymes [7,44].

From a nutritional and industrial perspective, these findings support fermentation as a promising technology for developing functional foods, sourdough baked goods, and gluten-free symbiotic beverages with potentially improved nutrient accessibility. When comparing this evidence with the scientific literature, the substantial improvement in mineral accessibility associated with phytate degradation is consistent with research that observed this same pattern of increased bioaccessibility in the fermentation of other Andean pseudocereals, such as quinoa and kañiwa, supporting fermentation as a promising biotechnological strategy for improving nutrient accessibility and reducing antinutritional factors [7,44,55].

Despite the potential of germination, extrusion, roasting, and fermentation to modify nutrient digestibility and bioaccessibility, their industrial implementation presents several challenges [16,49]. Variability among kiwicha ecotypes and raw material batches may affect processing consistency and product quality, requiring standardized raw material characterization and process control [3,7]. Moreover, operational costs associated with energy consumption, processing time, and equipment adaptation may limit the feasibility of scaling up these technologies, particularly for small- and medium-sized food manufacturers [36]. Therefore, future studies should integrate pilot-scale validation, techno-economic assessments, and product quality evaluations to determine whether improvements observed under laboratory conditions can be consistently reproduced in industrial food production [6,36].

## 7. Health Implications

The in vitro gastrointestinal digestion of kiwicha reveals complex interactions in which matrix structure and processing technologies modulate the bioaccessibility of its components [6,15]. As detailed in Table 5, the intestinal phase contributes to the release of low-molecular-weight peptide fractions (<5 kDa and <1 kDa) following proteolysis by pepsin and pancreatin, with reported in vitro protein digestibility values of up to 79.18% [6,18,29]. Some of these peptide fractions have exhibited inhibitory activity against enzymes such as α-amylase, ACE, or DPP-IV under in vitro experimental conditions. However, these enzyme-inhibition results indicate biochemical bioactivity and should not be interpreted as evidence of antidiabetic or antihypertensive efficacy. Moreover, the bioaccessibility of these peptides does not demonstrate their intestinal absorption, systemic bioavailability, or presence at biologically effective concentrations in vivo.

Concerning the carbohydrate matrix, starch digestibility is highly dependent on processing methods: moist heat methods such as boiling induce complete gelatinization, which facilitates rapid enzymatic hydrolysis (67.41%), whereas processes such as dry heat (whole-grain toasting) or bioprocessing (germination) generate robust steric barriers that restrict the accessibility of pancreatic *α*-amylase [15,56]. In kiwicha-containing breads and pasta, reductions in the hydrolysis index and estimated glycemic index have been reported under specific processing conditions [18,57,58]. However, these estimated values should not be interpreted as evidence of an equivalent reduction in the in vivo postprandial glycemic response, which requires direct evaluation in human studies. Gastrointestinal digestion can also increase the release or extractability of phenolic compounds and the measured antioxidant capacity of the intestinal digestate [18]. Nevertheless, increased phenolic release and antioxidant activity measured using chemical assays such as ORAC, FRAP, ABTS, or DPPH do not demonstrate intestinal absorption, systemic bioavailability, or antioxidant effects in humans. Finally, although kiwicha’s lipid fraction contains polyunsaturated fatty acids, phytosterols, and squalene, quantitative evidence concerning gastrointestinal lipolysis, micelle formation, and intestinal uptake remains limited [7,14].

Taken together, the available findings suggest that kiwicha possesses nutritional and functional attributes that could contribute to the development of foods with health-promoting properties. However, most of the evidence comes from in vitro studies and controlled experimental models. Therefore, future research should incorporate advanced digestion systems, intestinal absorption models, and clinical trials in humans to more accurately establish the physiological relevance of the compounds released during gastrointestinal digestion.

**Table 5:** Health implications associated with gastrointestinal digestion and the bioaccessibility of compounds found in kiwicha.

Health Area	Responsible Component	Type of Evidence	Key Findings	Proposed Mechanism	Potential Health Benefit	References
Bloodsugar control and diabetes	Intact starch vs. gelatinized starch	In vitro (Simulated GI digestion)	Toasting the whole grain limits starch hydrolysis to 15.5%; boiling increases it to 67.4%	Dry heat preserves the cell wall and generates Maillard compounds that act as a steric barrier against pancreatic α-amylase	Potential modulation of starch digestibility and postprandial glycemic response	[15]
	Complex matrix of sprouted kiwicha flour (fiber/protein)	In vitro (INFOGEST Protocol)	Reduction in the hydrolysis index (HI: 79.14) and estimated glycemic index (estimated GI: 83.16) relative to the control/reference in breads and pasta	The high concentration of protein and insoluble fiber disrupts the viscoelastic network, physically encapsulating the starch granules and reducing their enzymatic accessibility	Potential modulation of glycemic response in carbohydrate-rich processed foods	[8,18]
Cardiometabolic health (hypertension)	Bioactive peptides (<5 kDa) from glutelins and albumins	In vivo (hypertensive mouse models); In vitro	Significant reduction in systolic blood pressure; potent inhibition of the ACE enzyme	Peptides containing hydrophobic residues and proline block the active site of angiotensin-converting enzyme, inducing vasodilation	Antihypertensive activity observed in experimental models and potential cardiometabolic relevance	[59, 60]
Metabolic regulation (incretin effect)	Bioactive peptides derived from proteolysis	In vitro (enzymatic and cellular)	Strong inhibition of dipeptidyl peptidase-IV (DPP-IV) following gastrointestinal digestion	The peptides bind to the hydrophobic S1 site of DPP-IV, blocking its action and potentially influencing incretin activity	Potential relevance to incretin-related glucose regulation	[60, 61]
Gut health and anti-inflammation	Protein hydrolysates (KPHs)	In vitro (Caco-2 cell line)	Reduction of pro-inflammatory cytokines and restoration of epithelial homeostasis	Modulation and direct inhibition of the intracellular signaling cascade mediated by the NLRP3 inflammasome and NF-κB	Potential anti-inflammatory and intestinal epithelial protective activity	[62, 63]
Microbiome health (microbiota)	Insoluble and soluble dietary fiber (SDF)	In vitro (fecal fermentation)/In vivo	Substantial production of SCFAs (butyrate, propionate, acetate) and population modulation	The indigestible fiber matrix serves as a fermentable substrate for lactobacilli and bifidobacteria, inducing prebiotic metabolism in the colon	Potential relevance to colonic and metabolic health	[64, 65]
Antioxidant functionality	Free and bound phenolic compounds released	In vitro (INFOGEST static digestion)	The transition to the intestinal phase dramatically increases the release of phenols and antioxidant capacity (ORAC/FRAP)	Intestinal conditions and hydrolytic enzymes contribute to food-matrix disrupti of phytochemicals, increasing their bioaccessibility	Potential antioxidant functionality following gastrointestinal digestion	[15, 66]

Note: INFOGEST, standardized static in vitro gastrointestinal digestion protocol; HI, hydrolysis index; GI (glycemic index), estimated glycemic index; ACE, angiotensin-converting enzyme; DPP-IV, dipeptidyl peptidase IV; KPHs, kiwicha protein hydrolysates; NF-κB, nuclear factor kappa B; NLRP3, NOD-like receptor family pyrin domain-containing 3 inflammasome; SDF, soluble dietary fiber; SCFAs, short-chain fatty acids; ORAC, oxygen radical absorbance capacity; FRAP, ferric reducing antioxidant power; ROS, reactive oxygen species. The proposed health benefits are based on the experimental evidence reported in the cited studies and should not be interpreted as clinically proven effects in humans. Most findings derive from in vitro, animal, or preclinical studies and require confirmation through well-designed human clinical trials.

## 8. Challenges and Research Gaps

Research on the gastrointestinal digestion of kiwicha has advanced over the past decade; however, a critical review of the literature reveals significant limitations and scientific gaps that hinder the physiological validation of its potential health-related effects. The most pressing challenge is the excessive reliance on in vitro simulated digestion models and isolated assays (such as DPPH or ABTS antioxidant capacity tests), which have very limited physiological relevance given the complex dynamics of the human body [16,31]. Human intervention studies are particularly scarce, leaving the effects of dietary intake and the amounts needed to produce them uncertain [16]. This methodological gap prevents the conclusive extrapolation of “bioaccessibility” results to effective “bioavailability” [31]. Without robust clinical evidence to validate postprandial pharmacokinetics and pharmacodynamics, the available evidence remains insufficient to fully establish whether the antidiabetic, antihypertensive, and other metabolic activities observed in experimental studies translate into physiological benefits in humans [16,31,46].

Added to this limitation is the impact of high experimental variability, which hinders reproducibility and the advancement of knowledge. The literature reports significant heterogeneity stemming from the diversity of kiwicha genotypes and cultivars, the impact of agronomic conditions and geographic origin, as well as severe structural modifications induced by various thermal and non-thermal processing technologies [7,16]. This inherent variability is exacerbated by a critical lack of standardization in analytical methodologies, extraction protocols, and the units used to express results, which makes it impossible to conduct conclusive meta-analyzes or rigorous cross-sectional comparisons between studies [16]. Furthermore, in terms of digestive simulation, the use of static models predominates; while these models are reproducible, they fail to represent dynamic gastric emptying, peristalsis, or continuous pH changes, highlighting an urgent need to transition toward dynamic, multi-compartment gastrointestinal models [31].

The fate of kiwicha components in the distal stages of the digestive tract remains another major “black box” in current research. Most digestion protocols limit their analysis to the intestinal phase, showing limited integration of transepithelial absorption assays and systematically omitting the colonic fermentation phase [16,31]. Consequently, there is insufficient evidence regarding the interactions between the kiwicha matrix and the human gut microbiota [16]. It remains largely unknown whether and to what extent the high proportion of insoluble dietary fiber and phenolic compounds may modulate the ecology of the colonic microbiome, influence dysbiosis-related changes, or contribute to the production of short-chain fatty acids (SCFAs)—mechanisms that are fundamental to understanding the grain’s anti-inflammatory and metabolic regulatory effects [6,16]. Similarly, the literature lacks sufficient information on the bioaccessibility and bioavailability of the lipid fraction of kiwicha; despite its exceptional richness in high-value lipophilic compounds, such as squalene, tocopherols, and phytosterols, the dynamics of pancreatic lipolysis and the formation of mixed micelles during the digestion of this grain represent a critical scientific gap [7,31].

In the field of protein functionality, although it has been demonstrated that hydrolysates are released that inhibit key enzymes associated with metabolic syndrome (ACE, DPP-IV, α-amylase), there is a notable lack of studies dedicated to the precise identification, sequencing, and characterization of the specific bioactive peptides that survive gastrointestinal digestion [6,31]. To overcome these bottlenecks, it is a priority for future research to integrate advanced omics approaches that allow for tracking the complete metabolic fate of these compounds from their release to their systemic absorption [31,32]. Complementarily, invaluable opportunities are emerging for the application of artificial intelligence, data science, and predictive modeling (in silico) to simulate molecular docking between peptides and receptors, predict absorption, distribution, metabolism, and excretion (ADME) profiles, and assess potential allergenicity, thereby optimizing the selection of bioactive compounds prior to laboratory testing [16,31]. Overcoming these methodological limitations will allow for a more precise understanding of the mechanisms responsible for the physiological effects attributed to kiwicha and will facilitate the development of nutritional strategies and functional foods supported by more robust scientific evidence [8,16,31].

## 9. Future Perspectives

Future research on the gastrointestinal digestion of kiwicha should focus on the engineering of smart food matrices that optimize the bioaccessibility of its biologically valuable components using emerging technologies [22,67]. Future trends in food science suggest that the synergistic application of bioprocesses, such as germination and fermentation, together with advanced thermomechanical technologies such as extrusion, will allow for the intentional modulation of supramolecular interactions between starch, proteins, and dietary fiber [22,36,67]. This matrix structuring could facilitate controlled release kinetics during gastrointestinal transit, promoting high digestibility of macronutrients, the strategic release of phenolic compounds, and the production of bioactive peptides with potential inhibitory activity against enzymes linked to metabolic syndrome [31,36]. Furthermore, the adoption of predictive models and artificial intelligence tools could provide new opportunities for optimizing complex formulations and molecular interactions, supporting the design of foods with improved functional profiles [22].

From the perspective of precision nutrition, these technological innovations could support the use of kiwicha as an ingredient in the development of personalized functional foods designed for specific nutritional needs [22,36]. Kiwicha is naturally free of gluten and contains lysine, phytosterols, and squalene. These characteristics make it a useful ingredient to investigate foods designed for different dietary needs [14,36]. The formulation of complex matrices, such as nutraceutical pastes, synbiotic beverages, and extruded snacks, represents a potential opportunity for developing foods with relevance to metabolic health [8,36]. Similarly, these developments could contribute to the formulation of foods aimed at improving micronutrient intake during critical life stages (childhood, pregnancy, and old age) or supporting nutritional requirements related to muscle recovery and energy supply in sports nutrition [14,34,68].

Furthermore, integrating the study of kiwicha with the ecology of the gut microbiota represents an important research opportunity for elucidating its potential interactions with gut and metabolic health. A substantial proportion of the dietary fiber and insoluble phenolic compounds in this pseudocereal escape enzymatic digestion in the small intestine, reaching the colon where they may serve as fermentable substrates for the gut microbiota [36]. Future research should focus on how these indigestible fractions selectively modulate beneficial microbial populations, promoting the bioconversion of polyphenols and the synthesis of short-chain fatty acids (SCFAs), such as butyrate and propionate [31,36]. Understanding the mechanisms underlying this bidirectional interaction between the kiwicha matrix and the microbiome may provide new insights into potential applications related to gut and metabolic health, while clarifying the potential role of kiwicha in the gut–metabolism axis [31,36].

Therefore, advancing in this field will require multidisciplinary approaches integrating food science, nutrition, microbiology, molecular biology, and data science. Combining advanced digestive models, omics tools, and well-designed clinical interventions could enable a more robust mapping of kiwicha’s composition, its behavior during gastrointestinal digestion, and its potential physiological effects. This knowledge is essential to support the development of evidence-based functional foods and expand the use of this Andean pseudocereal in sustainable, health-oriented food systems.

Future studies should compare kiwicha varieties and processed foods using standardized methods that assess nutrient release, intestinal absorption, and fermentation of components that reach the colon. These findings could guide the development of foods that preserve the nutritional and cultural value of kiwicha. Research conducted with local producers and communities should also consider traditional uses, access to seeds, and the benefits of regional production. In this way, scientific advances may support the valorization of Andean foods and food sovereignty, while human studies determine whether the effects observed in experimental models translate into measurable health benefits.

## 10. Conclusions

Kiwicha is emerging as a nutritionally valuable pseudocereal whose nutritional quality is determined not only by its composition and food-matrix structure but also by the digestibility and bioaccessibility of its nutrients and bioactive compounds following gastrointestinal digestion. The evidence compiled in this review suggests that gastrointestinal digestion can promote the release of digestible proteins, peptide fractions, phenolic compounds, and other potentially bioactive components. Some of these digestion-derived compounds have shown antioxidant, ACE-inhibitory, glucose-modulating, and anti-inflammatory activities primarily under in vitro or preclinical conditions. However, these findings should not be interpreted as evidence of corresponding physiological effects in humans, for which direct evidence remains limited. Likewise, the structural characteristics of the starch–protein matrix may influence starch digestion kinetics, suggesting potential applications in the development of foods with modulated starch digestibility rather than demonstrated metabolic benefits.

Among the processing technologies investigated, germination is one of the most consistently studied approaches and may contribute to increased mineral bioaccessibility, phenolic compound release, GABA accumulation, and protein digestibility under experimental conditions through the activation of endogenous enzymes and reduction of antinutritional factors. Extrusion, depending on processing conditions, may modify protein digestibility and facilitate the development of gluten-free foods, whereas roasting has been associated with slower in vitro starch digestion and changes in antioxidant bioaccessibility. Fermentation may also influence mineral bioaccessibility and peptide generation, while conventional cooking can increase starch digestibility but may simultaneously promote losses of water-soluble bioactive compounds. These processing-related effects are strongly dependent on experimental conditions and should not be directly extrapolated to physiological or health benefits in humans.

Despite these promising findings, several important research gaps remain. Most available evidence relies on static in vitro digestion models, whereas information regarding intestinal absorption, lipid bioaccessibility, bioactive peptide metabolism, and long-term physiological effects is still limited. Therefore, future research should prioritize: (i) the integration of standardized INFOGEST digestion protocols with advanced intestinal absorption models; (ii) comprehensive characterization of bioactive peptides and lipid-derived metabolites generated during digestion; (iii) optimization of processing technologies to maximize nutrient bioaccessibility while preserving functional compounds; (iv) clinical validation of the potential metabolic and health-related effects of kiwicha-based foods; and (v) the development of innovative functional foods that take advantage of the unique structural and digestive characteristics of this Andean pseudocereal. Addressing these priorities will strengthen the scientific foundation for the industrial valorization of kiwicha and support its integration into sustainable, health-oriented food systems.

## Figures and Tables

**Table 1:** Relationships between structural components of the kiwicha food matrix, digestive mechanisms, and gastrointestinal digestion outcomes.

Structural Component	Role in the Food Matrix	Implications for Gastrointestinal Digestion	References
Starch granules	Small polygonal granules with low amylose content (~5–7%) and predominance of amylopectin.	Their relatively high surface area may favor enzymatic interaction; however, accessibility to α-amylase depends on matrix integrity and the extent of starch gelatinization.	[14,15]
Protein network	Storage proteins closely surround starch granules and can form aggregated networks during processing.	The protein network may restrict starch swelling and α-amylase accessibility, whereas proteolysis progressively disrupts this barrier and facilitates nutrient release.	[15,22]
Fiber structure	Cell-wall components and non-starch polysaccharides contribute to the physical integrity of the matrix.	Intact structures may limit enzyme penetration and delay the release of starch, proteins, and matrix-associated compounds.	[7,14,15,16]
Lipid distribution	Lipids are mainly associated with the germ and may interact with starch and proteins within the matrix.	Matrix interactions may influence substrate accessibility; however, direct evidence on kiwicha lipolysis, micelle formation, and intestinal absorption remains limited.	[7,15,22]

**Table 2:** Classification of experimental evidence supporting digestive and biological outcomes associated with *Amaranthus caudatus* L.

Evaluated Outcome	In Vitro Digestion/Acellular Assays	Cellular Models	Animal Studies	Human Studies	References
Proteindigestibility	Protein hydrolysis and digestibility have been evaluated using simulated gastrointestinal digestion of grains and protein fractions. Processing, including germination, malting, roasting, and extrusion, may modify digestibility depending on processing conditions	No direct evidence identified	Limited *A. caudatus*-specific evidence identified in the literature considered	No direct evidence identified	[3,19,29]
Peptide release and stability	Simulated gastrointestinal digestion with pepsin and pancreatin promotes protein hydrolysis and the release of low-molecular-weight peptide fractions (<5 kDa). Their persistence after simulated digestion provides evidence of digestive stability/bioaccessibility but not intestinal absorption	No direct evidence identified	No direct evidence identified	No direct evidence identified	[29,31]
ACE-inhibitory activity	ACE-inhibitory activity has been reported for enzymatically generated protein hydrolysates and peptide fractions under in vitro conditions. These findings indicate bioactivity but do not demonstrate systemic antihypertensive effects	No direct evidence identified	Antihypertensive responses have been reported in animal models receiving *A. caudatus*-derived preparations; these findings provide in vivo support but cannot be directly extrapolated to humans	No direct evidence identified for systemic antihypertensive effects specifically attributable to the ACE-inhibitory peptides identified in vitro	[31,35]
Antioxidant activity	Antioxidant activity has been reported using chemical assays, including DPPH, ABTS, FRAP, ORAC, and metal-chelating assays, in extracts and protein hydrolysates. These assays demonstrate chemical antioxidant capacity rather than physiological antioxidant effects	No direct evidence identified	Changes in oxidative-stress biomarkers and antioxidant enzyme activities have been reported in animal models receiving *A. caudatus*-derived preparations	No direct evidence identified	[7,12,29]

**Note:** Evidence was classified according to the experimental level at which each outcome was evaluated. “No direct evidence identified” indicates that no *A. caudatus*-specific evidence was identified in the literature considered in this review and should not be interpreted as evidence of absence. In vitro digestive or biochemical activity should not be interpreted as evidence of intestinal absorption, systemic bioavailability, or physiological efficacy in humans.

**Table 3:** Bioaccessibility of Nutrients and Bioactive Compounds in Kiwicha During Gastrointestinal Digestion.

Evaluated Component	Type of Kiwicha Sample	Processing Method or Technology Used	Digestion Model Used	Parameters Evaluated	Main Quantitative Results Reported	Nutritional or Functional Implications	References
Proteins and peptidos	Kiwicha protein isolate (KPI) and hydrolysates (KPHs)	Enzymatic hydrolysis (Bioprotease LA-660 at pH 8.0, 50 °C)	In vitro (digestion with pepsin-pancreatin)	In vitro protein digestibility, molecular weight profile determined by FPLC	Protein digestibility increased from 60.74% (defatted flour) to 77.97% in KPI and 79.18% in KPH5 (5 min). Peptides with a molecular weight <1 kDa were obtained	Generation of low-molecular-weight peptides with high in vitro digestibility and potential antioxidant activity	[5,29]
	Intact kiwicha protein	Pure simulated gastrointestinal digestion	Static in vitro	Inhibition of digestive enzymes associated with metabolic syndrome (IC_50_)	The intestinal digest generated peptide fractions (<5 kDa) with a potent inhibitory effect on α-amylase (IC 50 of 0.84 mg·mL^−1^)	Potential for the development of functional ingredients with in vitro enzyme-inhibitory activities relevant to glucose metabolism and ACE inhibition	[15,46]
Starch	Whole (intact) grain vs. homogenized suspension (slurry)	Heat treatment with moisture (boiling) vs. dry heat (toasting)	In vitro (Tamura model with gastric and intestinal phases)	Percentage of starch hydrolysis at equilibrium	The boiled intact grain reached 67.41% hydrolysis; the toasted intact grain was drastically limited to 15.50%.	Toasting was associated with substantially lower in vitro starch hydrolysis than boiling under the conditions studied	[15,39]
	Gluten-free sliced breads and pastries enriched with kiwicha flour	Controlled grain germination followed by baking or extrusion cooking	In vitro (INFOGEST 2.0 protocol)	Hydrolysis index (HI), estimated glycemic index (GI)	Bread made with germinated kiwicha reduced the HI to 79.14 and the GI to 83.16 (compared to 96.70 and 92.81 for the wheat control)	Potential strategy for modulating in vitro starch digestibility and estimated glycemic response in kiwicha-enriched food formulations	[1,8,18]
Lipids	Kiwicha grain/lipid fraction	No gastrointestinal lipid-digestion intervention specifically evaluated	No specific gastrointestinal lipid-digestion model identified	Pancreatic lipolysis, micellarization, and intestinal absorption	No quantitative kiwicha-specific data identified for lipid hydrolysis, micellarization, or intestinal absorption	Current evidence is primarily compositional; direct studies of pancreatic lipolysis, incorporation of fatty acids and phytosterols into mixed micelles, and intestinal absorption are required before lipid bioavailability or physiological effects can be established	[7,9,41]
Bioactive compounds	Whole grain and slurry	Boiled vs. roasted	In vitro (gastric and intestinal simulation)	Total phenolic content (TPC) released and antioxidant capacity (FRAP/ABTS)	The roasted whole grain released 311.73 mg GAE·100 g^−1^ DW after 360 min of intestinal digestion (compared with 143.93 mg GAE·100 g^−1^ DW for the boiled sample). The roasted slurry reached a maximum of 758.80 mg GAE·100 g^−1^ DW	Roasting and grinding were associated with increased release of phenolic compounds and antioxidant capacity during in vitro gastrointestinal digestion	[15,39,47]
	Breads formulated with sprouted kiwicha flour (SKF)	Bioprocessing (sprouting) and thermal baking	In vitro (INFOGEST 2.0 static protocol)	Bioavailability of TPC, GABA, and antioxidant capacity (ORAC)	In the final intestinal phase, the bread provided high levels of TPC (357.18 mg GAE·100 g^−1^ DW) and ORAC (109.11 µmol TE·g^−1^ DW). Bioaccessible GABA remained high in the intestinal digestate (18.5 mg·100 g^−1^ DW)	Retention of bioaccessible GABA and phenolic compounds after intestinal digestion, with potential relevance to antioxidant functionality	[8,18]

Note: KPI, kiwicha protein isolate; KPHs, kiwicha protein hydrolysates; FPLC, fast protein liquid chromatography; IC_50_, half maximal inhibitory concentration; HI, hydrolysis index; GI, estimated glycemic index; INFOGEST 2.0, standardized static in vitro gastrointestinal digestion protocol; TPC, total phenolic content; GABA, γ-aminobutyric acid; FRAP, ferric reducing antioxidant power; ABTS, 2,2′-azinobis(3-ethylbenzothiazoline-6-sulfonic acid) radical scavenging assay; ORAC, oxygen radical absorbance capacity; GAE, gallic acid equivalents; TE, Trolox equivalents; DW, dry weight. Quantitative results are expressed on a dry-weight basis where specified in the original studies.

## Data Availability

No new data were created or analyzed in this study. Data sharing is not applicable to this article.

## Abbreviations

The following abbreviations are used in this manuscript:

ABTS	2,2′-Azinobis(3-ethylbenzothiazoline-6-sulfonic acid)
ACE	Angiotensin-converting enzyme
ADME	Absorption, distribution, metabolism, and excretion
DH	Degree of hydrolysis
DIAAS	Digestible indispensable amino acid score
DPP-IV	Dipeptidyl peptidase IV
DPPH	2,2-Diphenyl-1-picrylhydrazyl
FPLC	Fast protein liquid chromatography
FRAP	Ferric reducing antioxidant power
GABA	γ-Aminobutyric acid
GAE	Gallic acid equivalents
GI	Glycemic index
HI	Hydrolysis index
HTST	High-temperature short-time
IC_50_	Half maximal inhibitory concentration
INFOGEST	Standardized static in vitro gastrointestinal digestion protocol
KPHs	Kiwicha protein hydrolysates
KPI	Kiwicha protein isolate
NF-κB	Nuclear factor kappa B
NLRP3	NOD-like receptor family pyrin domain-containing 3 inflammasome
ORAC	Oxygen radical absorbance capacity
PDCAAS	Protein digestibility-corrected amino acid score
RDS	Rapidly digestible starch
ROS	Reactive oxygen species
SCFAs	Short-chain fatty acids
SDF	Soluble dietary fiber
SKF	Sprouted kiwicha flour
TE	Trolox equivalents
TPC	Total phenolic content
TSP	Total soluble phenols
TSPC	Total phenolic compound content
